# Phytyl fatty acid esters in vegetables pose a risk for patients suffering from Refsum’s disease

**DOI:** 10.1371/journal.pone.0188035

**Published:** 2017-11-13

**Authors:** Stephanie Krauß, Lea Michaelis, Walter Vetter

**Affiliations:** Institute of Food Chemistry, University of Hohenheim, Stuttgart, Germany; University of New Orleans, UNITED STATES

## Abstract

Patients suffering from Refsum’s disease show mutations in the enzyme necessary for the degradation of phytanic acid. Accumulation of this tetramethyl-branched fatty acid in inner organs leads to severe neurological and cardiac dysfunctions which can even result in death. Thus, patients with Refsum’s disease have to follow a specific diet resigning foods with high levels of phytanic acid and *trans*-phytol like products from ruminant animals with a tolerable daily intake (TDI) of ≤ 10 mg/d. We recently reported the occurrence of phytyl fatty acid esters (PFAE, *trans*-phytol esterified with a fatty acid) in bell pepper with *trans*-phytol amounts of up to 5.4 mg/100 g fresh weight (FW). In this study we carried out *in vitro*-digestion experiments of PFAE with artificial digestion fluids. Our results demonstrate that PFAE actually are a source for bioavailable *trans-*phytol and thus add to the TDI. Eating only one portion of bell pepper (∼150 g) could therefore lead to exploitation of the TDI of up to 81%. Analysis of additional vegetable matrices showed that also rocket salad with up to 4.2 mg/100 g FW *trans*-phytol bound in PFAE represents a risk-relevant food for patients with Refsum’s disease and should therefore be taken into account.

## Introduction

**Fig 1:**
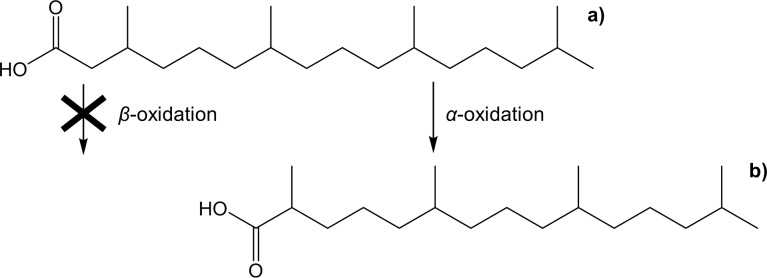
Structure of a) phytanic acid which cannot be degraded via *β*-oxidation and initially has to be converted into b) pristanic acid via *α*-oxidation before further metabolism.

Refsum’s disease–short for *heredopathia atactica polyneuritiformis*–is an inherited disorder of the lipid metabolism which affects the degradation of phytanic acid (3*R*/*S*,7*R*,11*R*,15-tetramethylhexadecanoic acid)–a tetramethyl-branched chain fatty acid (Fig 1A) [1–3]. Because of the methyl group located at C-3 phytanic acid cannot be degraded in the human body via *β*-oxidation like other fatty acids as the formation of the intermediate 3-ketoacyl-CoA is not possible [4,5]. Thus, phytanic acid has initially to be converted into pristanic acid (Fig 1B) through *α*-oxidation which moves the first methyl branch to C-2. Hence, pristanic acid can be degraded via *β*-oxidation [4,5].

Patients with Refsum’s disease show mutations in the enzyme phytanoyl-CoA-hydroxylase which results in the first step of *α*-oxidation of phytanic acid being blocked [6–8]. This leads to the accumulation of phytanic acid in blood plasma and inner organs like kidneys, liver or in the heart muscle [6,9,10]. Due to the high levels of phytanic acid (up to 50% of the total fatty acid content in the blood serum [2,11]) severe neurological as well as cardiac dysfunctions can occur which can even lead to death [1,5,7,11]. As the human body is not able to synthesize phytanic acid itself its accumulation is ascribed to exogenous origin, i.e. dietary intake [6,7]. The only possibility to treat Refsum’s disease lies in preventing the accumulation of phytanic acid through a special diet [5,6] and in very severe cases in plasma exchange [1,11]. The only source considered for the intake of phytanic acid is foods which are high in phytanic acid itself or its precursor *trans*-phytol (3,7*R*,11*R*,15-tetramethylhexadec-2E-en-1-ol) [4,12,13]. *trans*-Phytol is an isoprenoid alcohol which is mainly known as side chain of the chlorophyll molecule in which it is bound to the porphyrin ring via an ester bond and makes up one third of the molecular mass [13,14]. As the plant pigment chlorophyll is one of the most abundant organic substances on earth [15], one might think that the intake of *trans*-phytol might be especially high when consuming lots of green food because of the high chlorophyll contents [16]. However, the ester bond cannot be cleaved during human digestion, so that the chlorophyll phytol moiety is not bioavailable [9,14]. Only microorganisms occurring in the stomach of ruminants or in marine environments are able to release the side chain through their enzyme activity [6,13,14]. Therefore, patients with Refsum’s disease have to mainly avoid dairy products and meat from ruminants as well as fish fats [4–6,17]. Levels of free *trans*-phytol and phytanic acid in vegetables on the other hand are only low and not considered to be problematic [5,18]. As small amounts of phytanic acid can be degraded via the alternative way of *ω*-oxidation [1,2], the tolerable daily intake (TDI) of *trans*-phytol or better phytanic acid accounts for ≤ 10 mg/day for patients with Refsum’s disease [4,11].

Recently, we reported the occurrence of phytyl fatty acid esters (PFAE) in red and yellow bell pepper [19]. These relatively unknown substances consist of *trans*-phytol esterified with a fatty acid [20] and were to this point only described to occur in mosses [21], in the wax ester fraction of some edible oils like sunflower and olive oil [22,23] and in the cuticular waxes of some Amazonian plants [20]. Concentrations in the pulp of red and yellow bell pepper ranged between 0.9–11.2 mg/100 g fresh weight (FW) which corresponds with 0.4–5.4 mg *trans*-phytol [19]. However, it remained unclear, if cleavage of the PFAE into free *trans*-phytol and the fatty acid takes place during human digestion which would make PFAE a source of bioavailable *trans*-phytol [19].

In this study, we investigated the fate of PFAE in the human body by performing *in vitro*-digestion experiments to find an answer to this question. Therefore, we simulated the digestion process by treatment with an authentic PFAE standard and food samples (lipid extracts from bell pepper and rocket salad) with artificial digestion fluids. The authentic PFAE standard was synthesized following the isolation of natural *trans*-phytol from grass and subsequent enzymatic esterification with palmitic acid. Furthermore, we analyzed additional types of vegetables on a random basis regarding the occurrence of PFAE and the amounts of possibly released *trans*-phytol to find more relevant sources of maybe bioavailable *trans*-phytol for patients with Refsum’s disease.

## Materials and methods

### Samples

Samples were purchased between February and March 2017 in local supermarkets in Stuttgart, Germany. We analyzed the edible part (leaves) of rocket salad (n = 4, different packages), hot pepper (n = 4), carrots (n = 2), cucumber (n = 2), red grapes (n = 2; vines) as well as green and black olives (n = 2, each; 2,5 g per portion) on a random basis. Homogenization with a IKA A11 basic laboratory mill (IKA, Staufen, Germany) and further sample preparation was performed immediately after purchase.

As we could detect PFAE in yellow and red bell pepper but not in green ones in our preceding study we assumed that the occurence of PFAE could be linked to the chlorophyll breakdown and change of color during ripening [19]. Thus, we additionally analyzed tomatoes as they also count to the family of *Solanaceae* like bell pepper and change their color from green to red. However, we could not detect any PFAE in ripe tomatoes [19]. This indicated that the formation of PFAE could be ascribed to another source than chlorophyll breakdown which is why we randomized our sample selection.

### Chemicals

Palmitic acid (≥98%), NaCl (≥99%), glucuronic acid (>97%), KH_2_PO_4_ (>99.5%), uric acid (>98%) and pyridine (distilled prior use) were purchased from Fluka (Steinheim, Germany) while *n*-hexane and acetonitrile (both HPLC-grade) were from Th. Geyer (Renningen, Germany). KCl (>99%), KSCN (>99%), NaH_2_PO_4_∙H_2_O (>99%), CaCl_2_∙2H_2_O, pepsin, NH_4_Cl, NaHCO_3_ (≥99.5%) and *α*-D(+)-glucose (99%) were from Merck (Darmstadt, Germany). From Sigma-Aldrich (Steinheim, Germany) we ordered pancreatin, lipase Type II (activity 220 U/mg), albumin and *α*-amylase Type VI-B (activity 15.8 U/mg), which were isolated from *porcine pancreas*, each. Toluene (HPLC grade), NaOH (≥98%), Na_2_SO_4_ (≥99%) and bile salts (mixture of sodium cholate and sodium deoxycholate) were also from Sigma-Aldrich and urea (≥99.5%), KOH (>85%), ethanol (99.8%) as well as HCl (32%) were purchased from Carl Roth (Karlsruhe, Germany). Glucosamine-HCl was from Serva (Heidelberg, Germany), MgCl_2_∙6H_2_O was from Applichem (Darmstadt, Germany), *iso*-phytol (3,7,11,15-tetramethylhexadec-1-en-3-ol, >95%) was from TCI (Zwijndrecht, Belgium) and 5*α*-cholestane (98%) was from Acros Organics (Geel, Belgium). (N,O-bis-(trimethylsilyl)-trifluoroacetamide) (BSTFA) and trimethylchlorosilane (TMCS, 99:1 (*v/v*)) used for trimethylsilylation were purchased from Supelco (Bellefonte, PA, USA).

### Isolation of authentic *trans*-phytol from grass

Currently, phytol is only commercially available in form of a *cis*-/*trans*-phytol mixture which additionally is racemic on C-7 and C-11 [24]. In order to avoid any artefacts caused by the use of non-authentic phytol, the *trans*-phytol used for the esterification was isolated through direct saponification of 100 g grass according to Schröder *et al*. [24] Enrichment of *trans*-phytol from ∼0.9 g unsaponifiable matter of grass was performed with the counter current chromatography (CCC) system described by Hammann *et al*. [25] using coil 2 and 3 with a total volume of 236 mL. About 875 mg of unsaponifiable matter of grass was diluted in 10 mL (5 mL upper and lower phase, each) of the solvent system consisting of *n*-hexane/acetonitrile/toluene (4.25:4.25:1.5, *v/v/v*) and injected into the system which was operated in tail-to-head mode. Displacement of the stationary phase was 32 mL and stationary phase retention (S_f_) was 86%. Flow rate was set to 1 mL/min, the rotational speed of the centrifuge to 860 rpm and the UV/Vis-detector to λ = 290 nm. 24 fractions of 3 mL each were collected from 88 min to 130 min after injection. The solvent of each fraction was removed, the residue re-diluted in 1 mL of *n*-hexane and an aliquot was used for trimethylsilylation (Sample preparation for the determination of the *trans*-phytol moiety of phytyl fatty acid esters) and analysis by gas chromatography with mass spectrometry (Gas chromatography coupled to mass spectrometry (GC/MS)). The CCC fractions containing *trans*-phytol were combined and purified through solid phase extraction (SPE) on deactivated silica gel (Sample preparation for the determination of the *trans*-phytol moiety of phytyl fatty acid esters). Elution was carried out with different mixtures of *n*-hexane/ethyl acetate (Sample preparation for the determination of the *trans*-phytol moiety of phytyl fatty acid esters) whereas SPE fraction 4 (30 mL of ethyl acetate) provided 61,7 mg *trans*-phytol, which was adjusted to 1 mL of *n*-hexane and 22.6 mg were used for the synthesis of the phytyl palmitate standard (Synthesis of phytyl palmitate).

### Synthesis of phytyl palmitate

Preparation and cleanup of the phytyl palmitate (phytyl-16:0) standard used for the *in vitro*-digestion experiments was performed by enzymatic esterification based on Villeneuve *et al*. [26] and as described by Krauß *et al*. [19] The final product of phytyl palmitate was 23.8 mg (purity ∼98%).

### *In vitro*-digestion experiments

Artificial digestion fluids were prepared according to Heinlein and Buettner [27] who in turn based their experiment on the work of Oomen *et al*. [28] Constituents and concentrations as well as pH values were based on physiological conditions [28] (Table 1). For the *in vitro*-digestion we used 0.5 mg of phytyl palmitate as a pure standard solution as well as embedded in sample matrix, i.e. lipid extracts of bell pepper and rocket salad. The samples were placed in 100 mL round-bottom flasks, the solvent was evaporated, 5 mL of the artificial saliva was added and the mixture was stirred for 5 min at room temperature. The pH value (pH ∼ 6–7) was checked and 7.5 mL of the simulated gastric juice was added. The flasks were placed in a water bath of 37 ± 2°C for 2 h. After checking the pH value again (pH ∼ 1) and adding 15 mL of artificial intestinal juice as well as 5 mL of simulated bile, the mixture was stirred for additional 2 h at 37 ± 2°C (pH ∼ 8). The solutions were acidified with HCl and extracted three times with 10 mL of *n*-hexane. The *n-*hexane layers were combined, dried over Na_2_SO_4_ and filtrated into a 100-mL pear shaped flask. The volume of the solutions was adjusted to 1 mL each. Aliquots of 500 *μ*L were used for trimethylsilylation (Sample preparation for the determination of the *trans*-phytol moiety of phytyl fatty acid esters). The volume of the samples was adjusted to 1 mL of *n*-hexane and introduced to GC/MS analysis (Gas chromatography coupled to mass spectrometry (GC/MS)). In the same way, a blank sample was analyzed. Additionally, the samples (standard solution and lipid extracts) were treated in separate flasks with 32.5 mL of water instead of artificial digestive juices and stirred for 4 h 5 min at room temperature to rule out that cleavage of the PFAE took place without interaction with the enzymes. In a separate experiment, chlorophyll was treated with the simulated digestion fluids to confirm that *trans*-phytol is not set free from chlorophyll by the enzymes. Subsequently, we treated the pure phytyl palmitate standard as described above with the digestion fluids and took an aliquot of the solution after each step (1: saliva, 2: gastric juice, 3: intestinal juice and bile). Each *in vitro*-digestion experiment was carried out in duplicates.

**Table 1:** Composition of the digestion fluids.

	Saliva	Gastric juice	Intestinal juice	Bile
**Inorganic** **solution**	2 mL KCl (22.4 g/L)	3.7 mL KCl (22.4 g/L)	10 mL NaHCO_3_ (33.9 g/L)	8.54 mL NaHCO_3_ (33.9 g/L)
	1 mL KSCN (10.0 g/L)	3.1 mL NaCl (87.7 g/L)	8 mL NaCl (87.7 g/L)	3 mL NaCl (87.7 g/L)
	1 mL NaH_2_PO_4_∙2H_2_O (57.8 g/L)	2 mL NH_4_Cl (15.3 g/L)	2.5 mL KCl (22.4 g/L)	0.84 mL KCl (22.4 g/L)
	1 mL Na_2_SO_4_ (28.5 g/L)	1.8 mL CaCl_2_∙2H_2_O (22.2 g/L)	2 mL MgCl_2_∙6H_2_O (11.5 g/L)	0.01 mL HCl (32%)
	0.17 mL NaCl (87.7 g/L)	0.65 mL HCl (32%)	2 mL KH_2_PO_4_ (4.0 g/L)	
	0.09 mL NaOH (1 M)	0.6 mL NaH_2_PO_4_∙H_2_O (51.1 g/L)	0.018 mL HCl (32%)	
	***to 25 mL H***_***2***_***O***	***to 50 mL H***_***2***_***O***	***to 50 mL H***_***2***_***O***	***to 25 mL H***_***2***_***O***
**Organic** **solution**	1.6 mL urea (6.25 g/L)	2 mL glucose (32.5 g/L)	0.4 mL urea (6.25 g/L)	2 mL urea (6.25 g/L)
		2 mL glucuronic acid (1.0 g/L)		
		2 mL glucosamine-HCl (16.5 g/L)		
		1.36 mL urea (6.25 g/L)		
	***to 25 mL H***_***2***_***O***	***to 50 mL H***_***2***_***O***	***to 50 mL H***_***2***_***O***	***to 25 mL H***_***2***_***O***
**Additional** **substances**	7.25 mg *α*-amylase	0.1 g BSA	0.9 mL CaCl_2_∙2H_2_O (22.2 g/L)	0.5 mL CaCl_2_∙2H_2_O (22.2 g/L)
	0.75 mg uric acid	0.1 g pepsin	0.1 g BSA	0.09 g BSA
			0.3 g pancreatin	0.3 g bile salts
			0.5 g lipase	
**pH**	∼ 6.5	∼ 1.0	∼ 7.5–8.0	∼ 8.0

### Sample preparation for the determination of the *trans*-phytol moiety of phytyl fatty acid esters

Sample preparation was performed as described in detail by Krauß *et al*. [19] In brief, samples were extracted by focused open vessel microwave assisted extraction with the azeotropic mixture of cyclohexane and ethyl acetate (46:54, *w/w*) and PFAE were enriched by SPE on deactivated silica gel (20% water, *w/w*). The volume of the SPE fraction was adjusted to 1 mL of *n*-hexane. Instead of quantifying the intact PFAE, the *trans*-phytol moiety was determined after alkaline hydrolysis of the PFAE as the focus of this research rested on the amount of possibly released *trans*-phytol after digestion. This is the reason why no PFAE standard was added prior to SPE to not falsify the intensity of the GC/MS signal for *trans*-phytol after saponification. The recoveries of PFAE during SPE however, were determined separately and accounted for 102 ± 17% [19]. For saponification, an aliquot of SPE fraction 2 was placed into a 6 mL test tube, 5 *μ*g of *iso*-phytol was added as internal standard and the solvent was removed in a gentle stream of nitrogen. The residue was treated with 1.8 mL of ethanol and 0.2 mL of KOH (50% in water, *w/w*), the tubes were shaken vigorously and heated to 80°C for one hour. After cooling to room temperature 1 mL of distilled water was added and the unsaponifiable matter was extracted with 1 mL of *n*-hexane. For trimethylsilylation an aliquot of the extract (approx. 50 *μ*g of unsaponifiable matter) was treated with 50 *μ*L of BSTFA/TMCS (99:1, *v/v*) and 25 *μ*L of pyridine, before heating to 60°C for 30 min [25]. After removing the reagents, the residue was re-diluted in 1 mL of *n*-hexane. Before introducing the samples to GC/MS analysis (Gas chromatography coupled to mass spectrometry (GC/MS)) 3 *μ*g of 5*α*-cholestane was added as second internal standard to compensate for possible variances in the injection volume between subsequent injections.

### Gas chromatography coupled to mass spectrometry (GC/MS)

For determining the *trans*-phytol moiety of the PFAE (after silylation) a 6890/5973N MSD system (Agilent Technologies, Santa Clara, CA, USA) equipped with a split/splitless-injector was used as described in detail by Krauß *et al*. [19] Analyses were carried out in full scan as well as in selected ion monitoring (SIM) mode. For quantification, the ion with *m/z* 143.1 was used and its response factor was determined for both *iso*-phytol and *trans*-phytol in form of their trimethylsilyl ethers (TMS) from the chromatograms recorded in full scan mode. The limit of detection (LOD) in SIM mode ranged between ∼0.3–14 *μ*g/100 g FW and the limit of quantification (LOQ) between ∼1–46 *μ*g/100 g FW, depending on the vegetable analyzed.

The samples of the *in vitro* digestion experiments were analyzed using another 6890 GC system coupled to a 5973 MSD (Agilent Technologies, Santa Clara, CA, USA) as described before [19]. Parameters for analyses in SIM mode were slightly changed. Three time windows were chosen. Window 1 (6–15 min) covered the ions with *m/z* 123.1, 143.1 (phytyl-TMS) and *m/z* 117.1, 132.1, 313.3, 328.1 (16:0-TMS). Window 2 (15–24 min) included *m/z* 217.2, 353.2 (5*α*-cholestane), 239.2, 533.6 (phytyl-16:0), *m/z* 548.6, 253.2 (phytyl-17:0) and *m/z* 267.2, 562.6 (phytyl-18:0) while window 3 (24–41 min) contained *m/z* 296.2, 596.6 (phytyl-20:0), *m/z* 309.2, 604.6 (phytyl-21:0), *m/z* 618.6, 337.2 (phytyl-23:0) and *m/z* 351.2, 646.6 (phytyl-24:0). Additionally, all windows included *m/z* 123.1 and 278.2 (PFAE in general). CCC fractions (Isolation of authentic *trans*-phytol from grass) were also analyzed in full scan mode with the same GC/MS system.

## Results

### *In vitro*-digestion experiments

**Fig 2:**
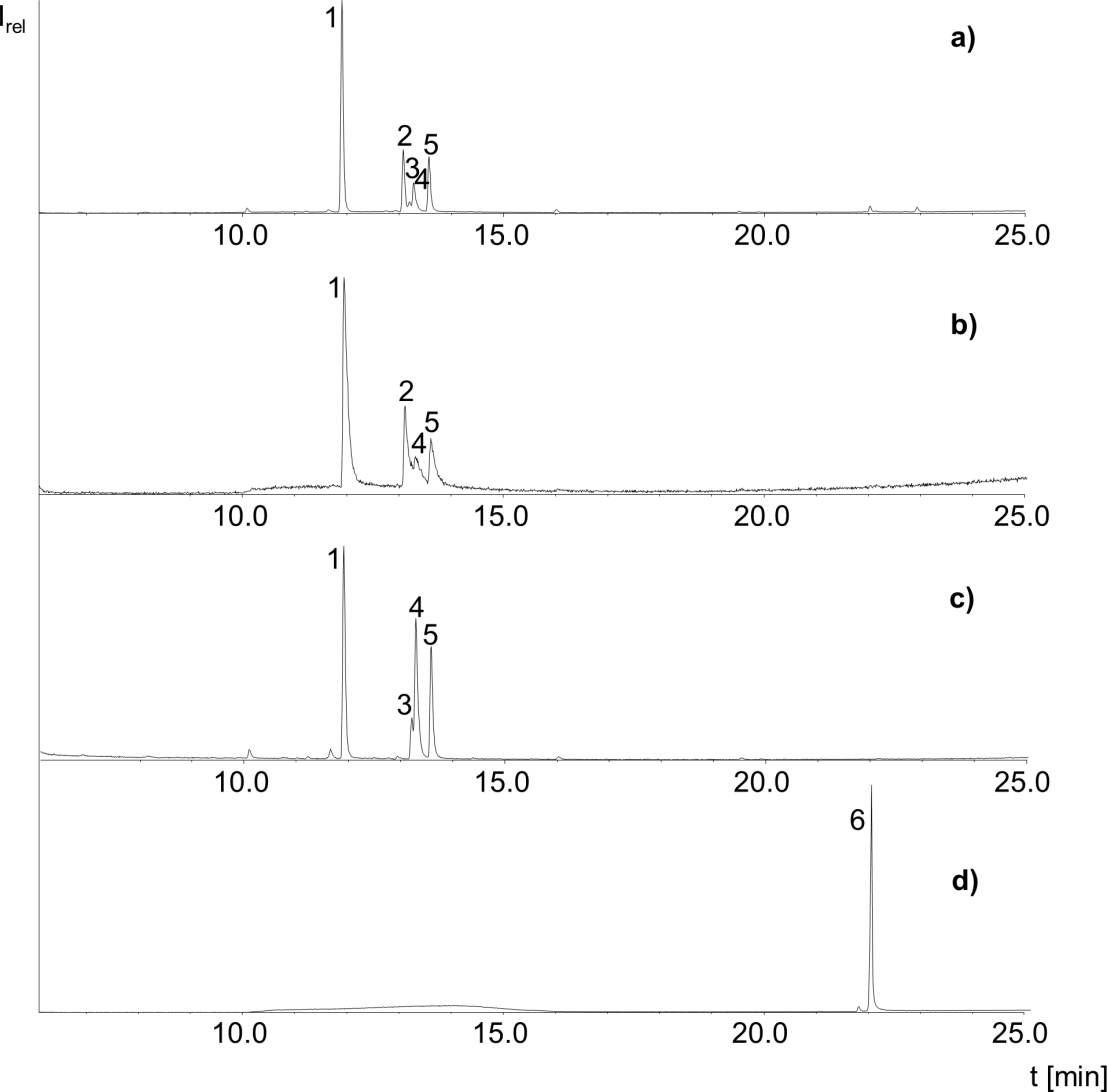
GC/MS full scan chromatogram of the samples after treatment with artificial digestion juices whereas samples consisted of (a) the pure phytyl palmitate standard (b) the phytyl palmitate standard embedded in the lipid extract of red bell pepper (c) the blank sample (d) the control sample of the phytyl palmitate standard, with identified peaks (1) 16:0-trimethylsilyl ester (TMS) (2) phytyl-trimethylsilyl ether (TMS), (3) 18:2-TMS, (4) 18:1-TMS, (5) 18:0-TMS, (6) phytyl-16:0.

Treatment of the synthesized phytyl palmitate standard with artificial digestion fluid generated free *trans*-phytol as well as the free palmitic acid whereas no intact phytyl palmitate could be detected (Fig 2A). Identification of *trans*-phytol in form of its trimethylsilyl ether (TMS) was possible by its characteristic GC/MS spectrum which is dominated by the ions with *m/z* 143.1 (base peak) and *m/z* 123.1 [29] and by comparing its retention time to a standard solution. In contrast, the control sample (without digestion fluids) showed solely the intact PFAE but no free *trans*-phytol or palmitic acid (Fig 2D). Same applies for the sample in which the phytyl palmitate standard was embedded in the lipid extract of bell pepper and rocket salad, respectively (Fig 2B). The remaining peaks in the GC/MS full scan chromatograms of the samples could be identified as additional fatty acids which were also detected in the chromatograms of the blank sample (Fig 2C). Their origin was ascribed to the enzymes that were used in the experiment. As the enzymes were isolated from porcine pancreas minute contamination with fatty acids is hardly avoidable. The experiment was repeated with chlorophyll under the same conditions. However, *trans*-phytol could neither be detected by GC/MS in full scan mode nor in SIM mode.

In an additional *in vitro*-digestion experiment with the phytyl palmitate standard we took an aliquot after step 1 (artificial saliva), 2 (simulated gastric juice) and 3 (intestinal juice and simulated bile). The GC/MS chromatograms of the first two stages did not show free *trans*-phytol, but we found a significant reduction of PFAE and a peak that could be identified as free *trans*-phytol in the chromatogram of the third step. Hence, *trans*-phytol was released from PFAE by intestinal juice/simulated bile but not with saliva and gastric juice. As artificial intestinal juice contains the enzyme lipase, this finding was not surprising and confirmed our presumption. It was also substantiated by the treatment of the PFAE standard with only the lipase at 37°C for 2 h which also showed cleavage of the ester bond.

### Determination of the phytol moiety of PFAE in different vegetables

*trans*-Phytol could be detected in all the analyzed samples after saponification of the SPE fraction containing PFAE. As free *trans*-phytol elutes into SPE fraction 4 as was verified with a standard solution, we could rule out that the *trans*-phytol identified in the samples derived from another source than PFAE. Correct determination of the *trans*-phytol moiety bound in PFAE was only possible after enrichment of PFAE by SPE and subsequent saponification. When determining the *trans*-phytol content after directly treating the lipid extract with alkali, the contents were about 2- to 10-fold lower than when quantification was carried out after PFAE enrichment [19]. Up to now we could not find an explanation for this phenomenon but attach great importance to it as otherwise the *trans*-phytol content is falsely determined to a lower than its actual value. Particularly for patients with Refsum’s disease this could be dangerous.

Amounts of *trans*-phytol derived from PFAE in carrots, cucumber, red grapes and green olives only ranged between 20 *μ*g to 90 *μ*g per 100 g FW (Table 2) and in black olives no *trans*-phytol was found at all. In contrast the contents in hot peperoni amounted to 2.4 mg phytol/100 g FW, in rocket salad to 4.2 mg/100 g FW and as reported earlier, in red and yellow bell pepper [19] to even 0.4 mg to 5.4 mg/100 g FW (Table 2). No statistical outliers could be detected with the David-Hartley-Pearson test (α = 0.05) for the *trans*-phytol amounts in peperoni, rocket salad and bell pepper. Therefore, the contents of *trans*-phytol derived from PFAE found in the analyzed samples lie within its naturally occurring range.

**Table 2:** *trans*-Phytol contents derived from PFAE in different vegetables [mg/100 g FW] and max. tolerable portion size [kg] to fully exploit the TDI (10 mg/d).

Vegetable/fruit	*trans*-phytol content [mg/100 g]	Portion size [kg] ≡ 10 mg *trans*-phytol
cucumber	0.02–0.04	25.000
red grapes	0.05–0.07	13.514
green olives	0.09	11.364
carrot	0.02–0.06	15.625
hot pepper	0.7–2.4	0.495
rocket salad	2.2–4.2	0.235
bell pepper (red/yellow)	0.4–5.4	0.185

## Discussion and conclusion

The results obtained from the *in vitro*-digestion experiments verified that cleavage of PFAE into *trans*-phytol and the fatty acid actually seems to take place during human digestion. Also, the vegetable matrix did not affect the release of the *trans*-phytol moiety of PFAE as the chromatograms were similar in both cases which showed no phytyl palmitate but simultaneously free *trans*-phytol and free palmitic acid.

To verify that the PFAE broke down during *in vitro*-digestion we additionally compared the ratio of palmitic acid to stearic acid in the GC/MS chromatogram of the blank sample and the samples which increased from < 2:1 to 3:1. As we ascribed the occurrence of stearic acid to the used enzymes we concluded that the increase of the palmitic acid in contrast to stearic acid must derive from the reduction of the phytyl palmitate. In combination with the free *trans*-phytol detected in the chromatograms of the samples it became apparent that PFAE indeed are a source for bioavailable *trans*-phytol.

**Fig 3:**
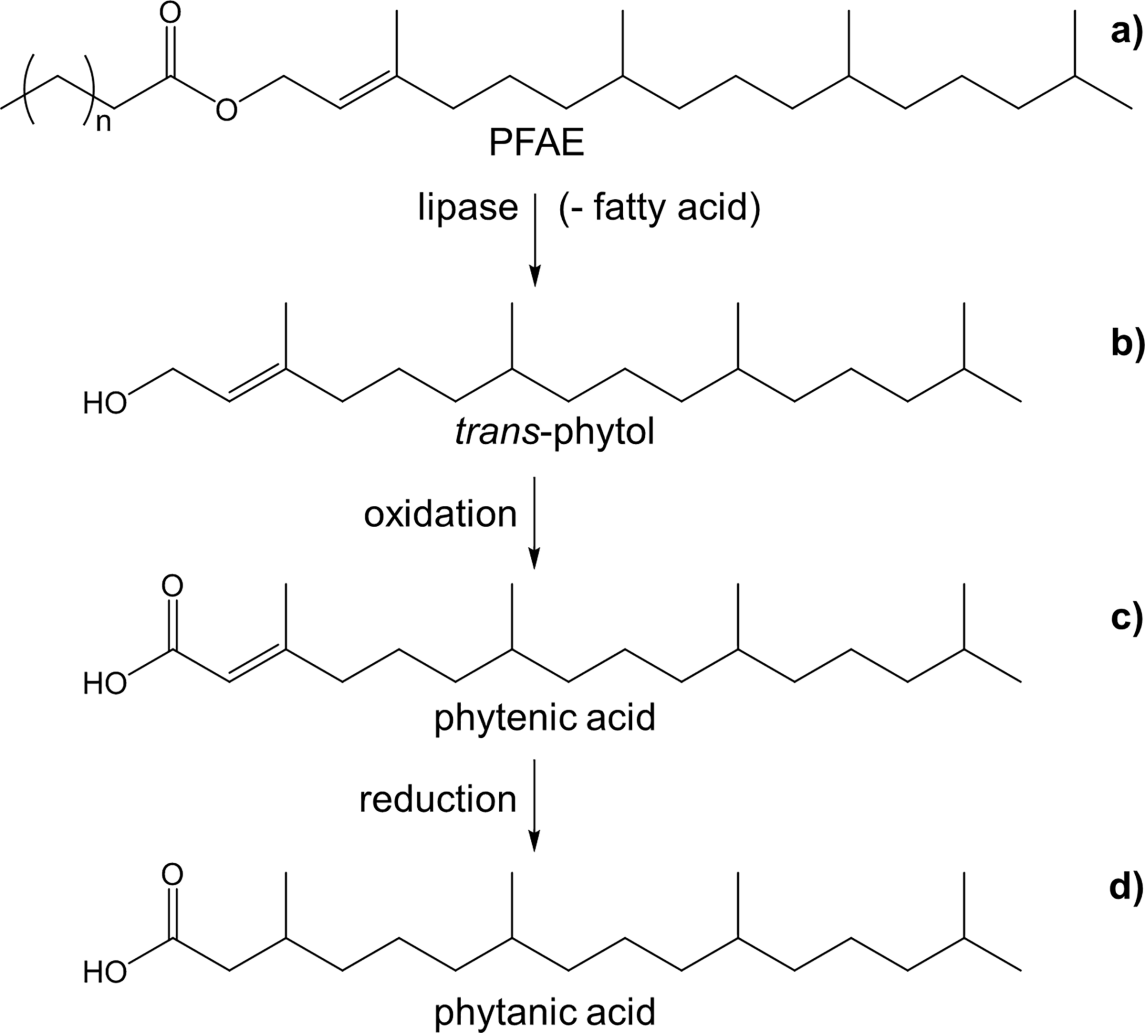
Schematic metabolism of PFAE in the human body with cleavage of PFAE (a) into the free fatty acid and free *trans*-phytol (b), which is then further metabolized by oxidation into phytenic acid (c) and finally by reduction of the double bond into phytanic acid (d) [6,30].

This means, consumption of PFAE leads to the release of *trans*-phytol (Fig 3A and 3B) through the lipase in the intestine. *trans*-Phytol can then be metabolized by oxidation into phytenic acid (Fig 3C), which is converted into phytanic acid by reduction of the double bond (Fig 3D) [6,30].

The fact that we could not detect free *trans*-phytol in the GC/MS chromatograms of digested chlorophyll indicated that the ester bond between the porphyrin ring and the phytyl side chain was not broken. This finding on the one hand confirmed that *trans*-phytol bound in chlorophyll is not bioavailable and on the other hand substantiated that the digestive conditions we used in this study actually are comparable to physiological conditions [9,14].

In our preceding study, we observed that no PFAE were present in the pulp of unripe green bell pepper but were detectable in amounts of up to 11.2 mg/100 g FW in the pulp of ripe red and yellow bell pepper [19]. This finding led us to the assumption that maybe the formation of PFAE could be linked to chlorophyll breakdown during ripening. Interestingly, PFAE were not detected in ripe tomatoes, which are also part of the family of *Solanaceae* like bell pepper and whose ripening is also accompanied by change in color. This result indicated that the esterification of phytol to fatty acids might be a separate process not linked to chlorophyll breakdown after all. The fact that unripe green olives contained PFAE but ripe black olives did not, emphasized this assumption. Considering its bioavailability out of PFAE, *trans*-phytol in different vegetables and fruits gains more importance as it actually contributes to the TDI (≤10 mg/d) for patients with Refsum’s disease. Regarding the maximally determined contents in 100 g of the particular vegetable, the TDI would be covered to less than 1% in case of cucumber, red grapes, green olives and carrot. Thus, these vegetables seem not to be risk-relevant for persons suffering from Refsum’s disease. Consumption of the same amount of hot pepper, rocket salad and red/yellow bell pepper however would contribute to up to 24%, 42% and 54% to the TDI, respectively. These amounts are alarming as with one average portion of red/yellow bell pepper (∼150 g) up to 81% of the TDI of *trans*-phytol could be reached. In addition, it cannot be excluded that samples are existing with higher *trans*-phytol content. As patients are allowed small amounts of *trans*-phytol/phytanic acid, the determined values would add to the *trans*-phytol that is taken in by their usual dietary habits which could easily lead to overstepping the TDI. Same applies for rocket salad. Additionally, we determined the portion size of each vegetable which would lead to a *trans*-phytol intake of 10 mg and thus to full exploitation of the TDI (Table 2). Even excessive consumption of cucumber, red grapes, olives and carrots would not lead to worrying intake of *trans*-phytol/phytanic acid as amounts of several kg of the particular vegetables/fruits are necessary to reach 10 mg which are highly unrealistic portion sizes (Table 2). Although hot pepper does contain up to 2.4 mg bioavailable *trans*-phytol per 100 g it still does not belong to the critical foods for patients with Refsum’s disease as the TDI would not be exploited until consuming about 500 g of hot pepper. As hot pepper is mainly used as spice ingredient and one piece only weighs about 15 g the contribution to the TDI is negligibly small.

Calculations show that only about 200 g of red/yellow bell pepper as well as rocket salad could be enough to reach the TDI of 10 mg *trans*-phytol/phytanic acid (Table 2). To make the numbers more concrete the *trans*-phytol content of 200 g of bell pepper equals the intake of phytanic acid by one glass of milk (100–200 mL) which had been identified as one of the most important nutritional sources of *trans*-phytol/phytanic acid [4,30]. As fruits and vegetables are considered to be practically free of phytanic acid [5] one could imagine that the diet of patients with Refsum’s disease might be richer in these foods. Our results only partly confirm that the consumption of fruits and vegetables does not add to the intake of phytanic acid. In case of tomato, cucumber, carrots, green olives and red grapes this might be true. However, others could unknowingly lead to the accumulation of phytanic acid as shown with this study and thus be of great risk. Based on our results we therefore highly recommend to avoid the consumption of red/yellow bell pepper and rocket salad or better to take the additional intake of *trans*-phytol/phytanic acid into account.

Further examination regarding the content of *trans*-phytol derived from PFAE will have to follow as the results obtained in this study only represent the data collected from just a few samples of each matrix. It is rather presumable that contents are scattered over a wider range like it was the case for bell pepper [19] and that higher amounts of *trans*-phytol could be ingested than the samples analyzed up to now reveal. Additionally, our results for bell pepper implied that the PFAE content differed regarding the season of harvest [19] which is why samples of different vegetables should be examined at different seasons to cover the full range of *trans*-phytol content possible in the particular matrix.

## References

[1] Weinstein R. Phytanic acid storage disease (Refsum’s disease): clinical characteristics, pathophysiology and the role of therapeutic apheresis in its management. J. Clin. Apheresis. 1999; 14: 181–184 10611628 10.1002/(sici)1098-1101(1999)14:4<181::aid-jca5>3.0.co;2-z

[2] Wierzbick AS, Lloyd MD, Schofield CJ, Feher MD, Gibberd FB. Refsum’s disease: a peroxisomal disorder affecting phytanic acid α-oxidation. J. Neurochem. 2002; 80: 727–735 11948235 10.1046/j.0022-3042.2002.00766.x

[3] Steinberg D, Avigan J, Mize C, Eldjarn L, Try K, Refsum S. Conversion of U-C14-phytol to phytanic acid and its oxidation in heredopathia atactica polyneuritiformis. Biochem. Bioph. Res. Co. 1965; 19: 783–789 10.1016/0006-291x(65)90328-1 4158442

[4] Vetter W, Schröder M. Phytanic acid—a tetramethyl-branched fatty acid in food. Lipid Technol. 2011; 23: 175–178

[5] Brown PJ, Mei G, Gibberd FB, Burston D, Mayne PD, McClinchy JE, et al Diet and Refsum’s disease. The determination of phytanic acid and phytol in certain foods and the application of this knowledge to the choice of suitable convenience foods for patients with Refsum’s disease. J. Hum. Nutr. Diet. 1993; 6: 295–305

[6] van den Brink D M, Wanders RJA. Phytanic acid: production from phytol, its breakdown and role in human disease. Cell. Mol. Life Sci. 2006; 63: 1752–1765 doi: 10.1007/s00018-005-5463-y 16799769 10.1007/s00018-005-5463-y PMC11136310

[7] Steinberg D, Mize CE, Avigan J, Fales HM, Eldjarn L, Try K, et al Studies on the metabolic error in Refsum’s disease. J. Clin. Invest. 1967; 46: 313–322 doi: 10.1172/JCI105533 4164676 10.1172/JCI105533 PMC297052

[8] van den Brink D. M., Brites P, Haasjes J, Wierzbicki AS, Mitchell J, Lambert-Hamill M, et al Identification of PEX7 as the second gene involved in Refsum disease. Am. J. Hum. Genet. 2003; 72: 471–477 12522768 10.1086/346093 PMC379239

[9] Baxter JH. Absorption of chlorophyll phytol in normal man and in patients with Refsum’s disease. J. Lipid Res. 1968; 9: 636–641 4177872

[10] Stoffel W, Kahlke W. The transformation of phytol into 3,7,11,15-tetramethylhexadecanoic (phytanic) acid in heredopathia atactica polyneuritiformis (Refsum’s syndrome). Biochem. Bioph. Res. Co. 1965; 19: 33–36

[11] Masters-Thomas A, Bailes J, Billimoria JD, Clemens ME, Gibberd FB, Page NGR. Heredopathia atactica polyneuritiformis (Refsum’s disease) 1. Clinical features and dietary management. J. Hum. Nutr. 1980; 34: 245–250 6157716 10.3109/09637488009143444

[12] Steinberg D, Herndon JH Jr, Uhlendorf BW, Mize CE, Avigan J, Milne GWA. Refsum’s disease: nature of the enzyme defect. Science. 1967; 156: 1740–1742 4180573 10.1126/science.156.3783.1740

[13] Patton S, Benson AA. Phytol metabolism in the bovine. Biochim. Biophys. Acta. 1966; 125: 22–32 5968593 10.1016/0005-2760(66)90140-8

[14] van den Brink DM, van Miert JNI, Dacremont G, Rontani J-F, Jansen GA, Wanders RJA. Identification of fatty aldehyde dehydrogenase in the breakdown of phytol to phytanic acid. Mol. Genet. Metab. 2004; 82: 33–37 doi: 10.1016/j.ymgme.2004.01.019 15110319 10.1016/j.ymgme.2004.01.019

[15] Scheer H. Chlorophyll breakdown in aquatic ecosystems. Proc. Natl. Acad. Sci. U. S. A. 2012; 109: 17311–17312 doi: 10.1073/pnas.1214999109 23071330 10.1073/pnas.1214999109 PMC3491517

[16] Ma L, Dolphin D. The metabolites of dietary chlorophylls. Phytochem. 1999; 50: 195–202

[17] Baldwin EJ, Harrington DJ, Sampson B, Feher MD, Wierzbicki AS. Safety of long-term restrictive diets for peroxisomal disorders: vitamin and trace element status of patients treated for adult Refsum Disease. Int. J. Clin. Pract. 2016; 70: 229–235 doi: 10.1111/ijcp.12770 26799636 10.1111/ijcp.12770

[18] Roca-Saavedra P, Marino-Lorenzo P, Miranda JM, Porto-Arias JJ, Lamas A, Vazquez BI, et al Phytanic acid consumption and human health, risks, benefits and future trends: A review. Food Chem. 2017; 221: 237–247 doi: 10.1016/j.foodchem.2016.10.074 27979198 10.1016/j.foodchem.2016.10.074

[19] Krauß S, Hammann S, Vetter W. Phytyl fatty acid esters in the pulp of bell pepper (Capsicum annuum). J. Agric. Food Chem. 2016; 64: 6306–6311 doi: 10.1021/acs.jafc.6b02645 27458658 10.1021/acs.jafc.6b02645

[20] Pereira AS, Siqueira DS, Elias VO, Simoneit BR, Cabral JA, Aquino Neto FR. Three series of high molecular weight alkanoates found in Amazonian plants. Phytochem. 2002; 61: 711–719 10.1016/s0031-9422(02)00348-5 12423894

[21] Suga T, Aoki T. The first naturally occuring phytyl esters and hexane soluble non-volatiles from leaves of Fatsia Japonica. Phytochem. 1974; 13: 1623–1624

[22] Reiter B, Lorbeer E. Analysis of the wax ester fraction of olive oil and sunflower oil by gas chromatography and gas chromatography-mass spectrometry. J. Am. Oil Chem. Soc. 2001; 78: 881–888

[23] Biedermann M, Haase-Aschoff P, Grob K. Wax ester fraction of edible oils. Eur. J. Lipid Sci. Technol. 2008; 110: 1084–1094

[24] Schröder M, Lutz NL, Vetter W. GC/MS and 1H-NMR analysis of phytanic acid synthesized from natural trans-phytol and a synthetic phytol standard. Chromatographia. 2014; 77: 379–385

[25] Hammann S, Englert M, Muller M, Vetter W. Accelerated separation of GC-amenable lipid classes in plant oils by countercurrent chromatography in the co-current mode. Anal. Bioanal. Chem. 2015; 407: 9019–9028 doi: 10.1007/s00216-015-9068-5 26438473 10.1007/s00216-015-9068-5

[26] Villeneuve P, Turon F, Caro Y, Escoffier R, Baréa B, Barouh B, et al Lipase-catalyzed synthesis of canola phytosterols oleate esters as cholesterol lowering agents. Enzyme Microb. Tech. 2005; 37: 150–155

[27] Heinlein A, Buettner A. Monitoring of biotransformation of hop aroma compounds in an in vitro digestion model. Food Funct. 2012; 3: 1059–1067 doi: 10.1039/c2fo30061c 22740026 10.1039/c2fo30061c

[28] Oomen AG, Rompelberg CJM, Bruil MA, Dobbe CJG, Pereboom DPKH, Sips AJAM. Development of an in vitro digestion model for estimating the bioaccessibility of soil contaminants. Arch. Environ. Contam. Toxicol. 2003; 44: 281–287 doi: 10.1007/s00244-002-1278-0 12712286 10.1007/s00244-002-1278-0

[29] Schröder M, Lehnert K, Hammann S, Vetter W. Dihydrophytol and phytol isomers as marker substances for hydrogenated and refined vegetable oils. Eur. J. Lipid Sci. Technol. 2014; 116: 1372–1380

[30] Vetter W, Schröder M. Concentrations of phytanic acid and pristanic acid are higher in organic than in conventional dairy products from the German market. Food Chem. 2010; 119: 746–752

